# The Potential Synergic Effect of a Complex Pattern of Multiple Inherited Genetic Variants as a Pathogenic Factor for Ovarian Dysgenesis: A Case Report

**DOI:** 10.3389/fendo.2020.540683

**Published:** 2020-09-25

**Authors:** Alessandro Cattoni, Alice Spano, Anna Tulone, Annalisa Boneschi, Nicoletta Masera, Silvia Maitz, Anna Maria Di Blasio, Luca Persani, Fabiana Guizzardi, Raffaella Rossetti

**Affiliations:** ^1^Department of Pediatrics, Azienda Ospedaliera San Gerardo, Università degli Studi di Milano Bicocca, Fondazione Monza e Brianza per il Bambino e la Sua Mamma, Monza, Italy; ^2^Department of Gynecology and Obstetrics, Azienda Ospedaliera San Gerardo, Fondazione Monza e Brianza per il Bambino e la Sua Mamma, Monza, Italy; ^3^Molecular Biology Laboratory, Istituto di Ricovero e Cura a Carattere Scientifico Istituto Auxologico Italiano, Cusano Milanino, Italy; ^4^Department of Endocrine and Metabolic Diseases and Lab of Endocrine and Metabolic Research, Istituto Auxologico Italiano, Milan, Italy; ^5^Department of Clinical Sciences and Community Health, University of Milan, Milan, Italy

**Keywords:** primary ovarian insufficiency (POI), gonadal dysgenesis (GD), antimullerian hormone (AMH), FIGLA gene, NOBOX gene, NR5A1 gene, fertility, gene variants

## Abstract

Non-syndromic primary ovarian insufficiency due to ovarian dysgenesis in 46,XX patients is an uncommon finding in the general population, even though several monogenic variants have been reported as causative factors. Here, we describe a 15-year-old patient diagnosed with gonadal dysgenesis possibly due to the interaction of three potentially pathogenic variants of genes involved in ovarian maturation, namely *factor in the germline alpha* (*FIGLA*), *newborn ovary homeobox-encoding* (*NOBOX*) and *nuclear receptor subfamily 5 group A member 1* (*NR5A1*). We also describe a different degree of residual ovarian function within the proband's family, whose female members carry one to three demonstrated variations in the aforementioned genes in a clinical *spectrum* potentially dependent on the number of alleles involved. Our results support the hypothesis that the severity of the clinical picture of the proband, resulting in complete ovarian dysgenesis, may be due to a synergic detrimental effect of inherited genetic variants.

## Introduction

Primary ovarian insufficiency (POI) is defined as the combination of amenorrhea reported for at least 4 months and enhanced levels of follicle-stimulating hormone (FSH) within the post-menopausal range at two or more subsequent assessments in women younger than 40 years (1). Amenorrhea can be either primary—about 10% of non-iatrogenic cases—or secondary (2). POI can present as an isolated condition, or it can fall within the clinical setting of a defined or undefined syndrome (3). Overall, it affects 1:10,000 women before age of 20, 1:1,000 women before the age of 30, and 1:100 women before the age of 40 years (4).

Several risk factors may contribute to the pathogenesis of POI, with both genetic and non-genetic causes being potentially involved. However, in 50–80% of cases, the underlying mechanism is unknown, and POI is classified as idiopathic. Iatrogenic interventions (i.e., pelvic surgery and anti-cancer treatment), autoimmune conditions, infectious agents (e.g., viral oophoritis) and environmental disruptors (e.g., exposure to 1-bromopropane) can all be listed among the non-genetic factors harmful to ovarian function (5).

Either chromosomal aberrations or monogenic disorders are identified in about 10% of patients diagnosed with POI (6). In addition, it is highly likely that a large percentage of idiopathic POIs are caused by yet unidentified genetic mutations.

Genetic disorders, such as chromosomal abnormalities and monogenic or polygenic mutations involving genes located either on autosomal chromosomes or on the X chromosome, have been described both in syndromic and non-syndromic cases of POI (5). In particular, chromosome aberrations alone (i.e., Turner's syndrome, triple X syndrome, fragile X syndrome and Robertsonian translocations) cause ~9 % of all POI (1, 3, 4). In addition, as the human reproductive phenotype is the result of the structured interaction of the products of several genetic sequences, different combinations of genetic variations may lead to POI.

Although there has been a significant effort in sequencing several POI candidate genes, only few coding mutations have been described so far, which is consistent with the low incidence of this condition. These genes mostly include transcription factors involved in female gonadal embryonal maturation and development. Currently, mutations in *FSHR, LHCGR, NR5A1, NOBOX, FOXL2, FIGLA, BMP15, NANOS3*, and *STAG3* genes have all been formally validated as being causative of non-syndromic POI (5).

Ovarian dysgenesis can be regarded as the most severe clinical picture among those related to POI. In patients affected by this condition, the ovaries are degenerated and completely depleted of follicles before puberty, and only streak gonads are present (3). Ovarian dysgenesis is the most common finding in girls with Turner's syndrome. However, when it occurs in patients with normal sex chromosomes, it gives rise to the “46, XX pure gonadal dysgenesis.” The clinical phenotype of this condition includes primary amenorrhea, lack of breast development, hypoestrogenism, and raised gonadotropins (3, 7).

Clinical suspicion of POI should be raised whenever a girl presents with primary amenorrhea—defined as the absence of menses at the age of 15 years in the presence of normal secondary sexual development—or fails to initiate breast development by the age of 13 years, all conditions where biochemical and radiological investigations are highly recommended (7).

In the present study, we describe the case of a 15-year-old girl diagnosed with POI possibly due to a complex pattern of compound monogenic alterations.

## Case Description

The proband is a Caucasian female patient referred to our endocrine outpatient clinic at the age of 15.1 years due to lack of secondary sexual characteristics. The female members of her family had achieved menarche at a suitable age, and no family history consistent with pubertal delay was reported. As the patient's father had prematurely committed suicide, few data about the timing of his pubertal development were available at the time of consultation. The patient was the second child of non-consanguineous parents, and she was born at term following an uneventful pregnancy. She had achieved all the developmental motor and intellectual milestones at an appropriate age.

On physical examination, the proband was classified as Tanner stage B1PH3AH2 and did not present with any signs of estrogenization of genitalia or areolas.

From an auxological perspective, the patient had had a normal growth along the 25^th^ centile (WHO growth charts) until the age of 10 years, but she had subsequently experienced a progressive decrease in height SDS, with a growth velocity of 3.2 cm/years. This clinical picture was deemed consistent with constitutional delay of growth and pubertal development. On first examination, her stature was 152.0 cm (8^th^ centile, −1.41 SDS), while her BMI was 15.37 Kg/m^2^ (−2.31 SDS), with no weight gain or loss recently reported.

Baseline biochemical and radiological assessment showed normal thyroid function and prolactin levels and negative anti-transglutaminase antibodies, ruling out potential causative factors of pubertal delay. Insulin-like growth factor 1 (IGF-1) levels were within the reference range for chronological and bone age. Cortisol (8.2 μg/dL) and ACTH (42 pg/mL) were normal as well. Nonetheless, the patient presented with high levels of serum gonadotropins (LH 34 U/L, FSH 159.9 U/L) with undetectable estradiol (<5 pg/mL).

Hand and wrist X-ray revealed a remarkably delayed bone age (12.0 *vs*. a chronological age of 15.1 years), while pelvic ultrasound (US), performed to explore internal genitalia, showed a prepubertal uterus (body 1.1 cm, cervix 1.3 cm) with undetectable ovaries. Pelvic MRI confirmed the finding of infantile internal genitalia, with the typical occurrence of small, streak gonads. Dual X-ray absorptiometry (DXA) scan showed severely reduced bone mineral density (lumbar Z score: −3.9; total body Z score:−3.3). Negative anti-ovary antibodies and autoimmune screening tests (i.e., ANA, ENA, anti-thyroid autoantibodies), previous medical history and normal female karyotype (46,XX) ruled out both non-genetic causes of POI and chromosomal abnormalities.

As all the aforementioned findings, together with the psychological impact of severe pubertal delay, made it necessary to perform immediate pubertal induction, the patient was started on progressively increasing doses of transdermal 17-β-estradiol, which led to subsequent development of secondary sexual characteristics. After 24 months of treatment, she started to experience vaginal spotting. At that point, a new pelvic US showed a post-pubertal appearance of her uterus with thickened endometrial line. Thus, micronized progesterone was administered for 14 every 28 days in order to achieve regular menses.

Dual-energy X-ray absorptiometry (DXA scan), repeated 2 years after the start of hormone therapy, showed a remarkable improvement in both total body and lumbar densitometry (Z scores: −2.4 and −3.1, respectively).

**Figure 1:**
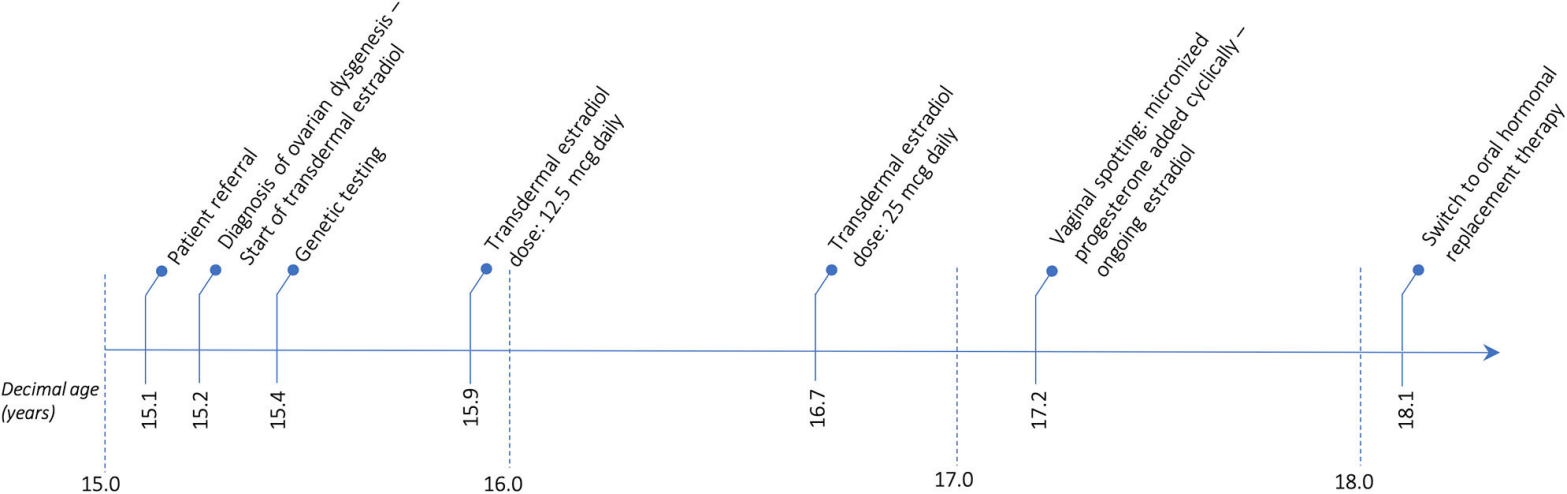
Timetable describing the sequence of the diagnostic and therapeutic interventions undertaken for the proband.

**Figure 2:**
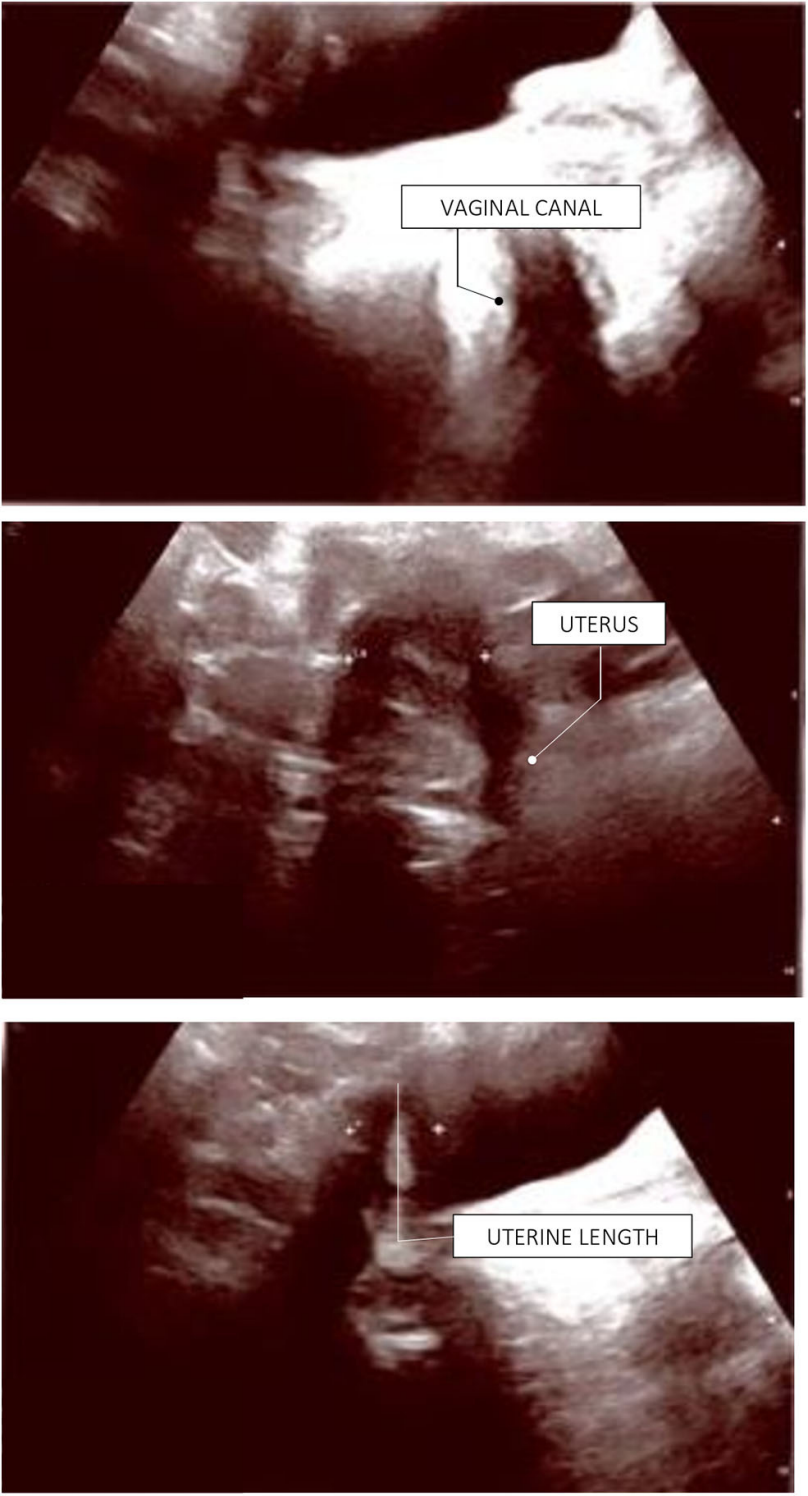
Proband's last pelvic ultrasound, performed at the end of the pubertal induction. Ovaries were not detected due to ovarian dysgenesis. The uterus has reached a mature post-pubertal shape and volume (9.5 mL). Endometrial thickness: 4 mm (secretive phase).

The patient has now achieved a complete pubertal development and has recently been switched to a combined therapy with oral 17ß-estradiol and dydrogesterone in order to improve the compliance to treatment. Her final height of 161.3 cm (39^th^ centile, −0.28 SDS) is entirely within the mid-parental target height. Figure 1 represents the timeline of all diagnostic and therapeutic interventions performed for the proband. Figure 2 represents the pictures captured from the most recently performed pelvic US.

Due to complaints of impaired self-esteem and perceived low levels of social interaction with peers, the patient has also been under the care of a psychologist since her initial diagnosis of POI. Hormonal treatment and subsequent development of secondary sexual features have helped the patient overcome her psychological issues. Currently, her self-esteem and own perception of physical attributes have remarkably improved.

### Genetic Analyses

Blood samples from both the proband and her relatives were collected after informed consent and analyzed by the IRCCS Istituto Auxologico Italiano, Laboratories of Endocrine and Metabolic Research and Molecular Biology. All coding and flanking intronic regions of a wide panel of genes potentially involved in POI were analyzed *via* Next Generation Sequencing. These included the *BMP15, FIGLA, FOXL2, FSHR, GDF9, NOBOX, NR5A1*, and *GALT* genes. The library was generated through Nextera Rapid Capture Custom Enrichment (Illumina). As a result, a complex pattern of genetic variations in the proband was detected. Firstly, we identified a rare frameshift homozygous variant within exon 2 of the *FIGLA* (*factor in the germline alpha*) gene [NM_001004311.3(FIGLA_v001):c.364del,NM_001004311.3 (FIGLA_i001):p.(Glu122Lysfs^*^45)], generating a truncated protein. Secondly, we found a novel heterozygous frameshift variant within exon 9 of the *NOBOX* (*newborn ovary homeobox-encoding*) gene [NM_001080413.3(NOBOX_v001):c.1626del, NM_001080413.3 (NOBOX_i001):p.(Phe543Serfs^*^7)], encoding for a truncated protein as well. Both deletions involved a well-known functional domain and were predicted to likely be disease-causing by a bioinformatic pathogenicity prediction tool (Table 1). Lastly, we detected a rare missense variant of the *NR5A1* (*nuclear receptor subfamily 5 group A member 1*) gene NM_004959.5(NR5A1_v001):c.1063G>A, NM_004959.5(NR5A1_i001):p.(Val355Met), which was predicted to be pathogenic by 5 out of 7 prediction tools available online (Table 1).

**Table 1:** Pathogenicity prediction for the three genic variants detected in the family described.

**Gene**	**Transcript**	**Sequence variation**	**AA change**	**InterPro domain**	**EXAC MAF^1^**	**Pathogenicity prediction**						
						**Mutation Taster^4^**	**Polyphen-2^5^**	**SIFT^6^**	**CADD^7^**	**Revel^8^**	**MetalR^9^**	**Mutation Assessor^10^**
FIGLA	NM_001004311.3	c.364del	p.Glu122Lysis*45	bHLH^2^	<0.01	Disease causing(1)	–	–	–	–	–	–
NOBOX	NM_001080413.3	c.1626del	p.Phe543Serfs*7	–	–	Disease causing(1)	–	–	–	–	–	–
NR5A1	NM_004959.5	c.1063G>A	p.Val355Met	LBD^3^	<0.01	Disease causing(0.99)	Probably Damaging(0.982)	Deleterious(0)	Likely benign(26)	Likely disease causing(0.816)	Damaging (0.953)	Medium (0.832)

1 *MAF, Minor Allele Frequency*.

2 *bHLH, basic Helix-Loop-Helix domain of DNA binding*.

3 *LBD, Ligand-Binding Domain at C-term of Nuclear receptors (NR)*.

4 *MutationTaster employs a Bayes classifier to eventually predict the disease potential of an alteration. The probability value is the probability of the prediction, i.e., a value close to 1 indicates a high “security” of the prediction*.

5 *PolyPhen-2 (Polymorphism Phenotyping v2) is a tool which predicts possible impact of an amino acid substitution on the structure and function of a human protein using straightforward physical and comparative considerations. PolyPhen-2 predicts the functional significance of an allele replacement from its individual features by Naïve Bayes classifier trained using supervised machine-learning. http://genetics.bwh.harvard.edu/pph2/. Adzhubei et al. (8)*.

6 *SIFT predicts whether an amino acid substitution affects protein function based on sequence homology and the physical properties of amino acids. SIFT can be applied to missense variants. https://sift.bii.a-star.edu.sg/. Vaser et al. (9)*.

7 *CADD is a tool that integrates multiple annotations into one metric for scoring the deleteriousness of single nucleotide variants. https://cadd.gs.washington.edu/. Rentzsch et al. (10)*.

8 *REVEL, Rare Exome Variant Ensemble Learner, is an ensemble method for predicting the pathogenicity of missense variants. It integrates scores from MutPred, FATHMM v2.3, VEST 3.0, PolyPhen-2, SIFT, PROVEAN, MutationAssessor, MutationTaster, LRT, GERP++, SiPhy, phyloP, and phastCons. Score range from 0 to 1 and variants with higher scores are predicted to be more likely to be pathogenic*.

9 *MetaLR uses logistic regression to integrate nine independent variant deleteriousness scores and allele frequency information to predict the deleteriousness of missense variants. Variants are classified as “tolerated” or “damaging”; a score between 0 and 1 is also provided and variants with higher scores are more likely to be deleterious*.

10 *Mutation Assessor predicts the functional impact of amino-acid substitutions in proteins using the evolutionary conservation of the affected amino acid in protein homologs. The prediction can be “neutral,” “low,” “medium,” and “high,” and the rank score is between 0 and 1 where variants with higher scores are more likely to be deleterious*.

In order to further assess the pathogenic role of these variants, the analysis was extended to the patient's first-degree relatives, namely her mother and sister. While the proband's mother was a heterozygous carrier of the *FIGLA* nucleotide deletion, her sister—aged 19 years at that time—harbored heterozygous variants in both *FIGLA* and *NR5A1* genes. Thus, it is likely that both sisters inherited the *NR5A1* missense variant from their father. All the identified variants of the *FIGLA, NOBOX*, and *NR5A1* genes were confirmed in the proband and assessed in her mother and sister by Sanger sequencing.

**Figure 3:**
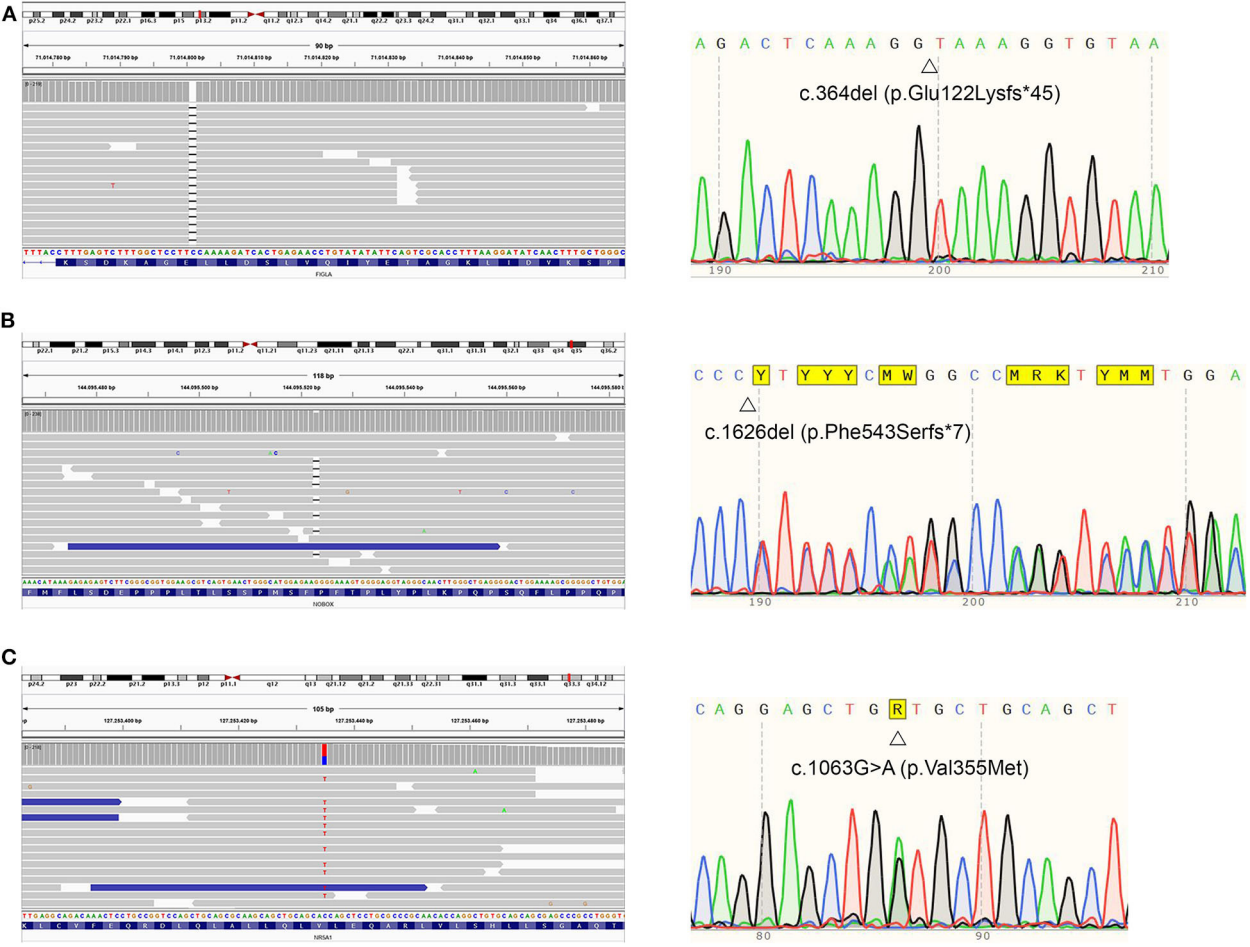
Proband's NGS profiles (right) and sequence (left) of *FIGLA, NOBOX*, and *NR5A1* genes. The identified variants c.364del **(A)**, c.1626del **(B)**, and c.1063G>A **(C)** are indicated.

Despite having non-consanguineous parents, the patient was homozygous for an extremely rare *FIGLA* variant. In order to explain this peculiarity, additional analyses were conducted (see Supplementary files for an in-depth explanation). Real-time PCR ruled out a deletion involving the long arm of chromosome 2. Furthermore, a microsatellite analysis was performed, and it excluded uniparental isodisomy (UPD) for chromosome 2. Nevertheless, we were not able to exclude segmental UPD, albeit rare. Figure 3 represents the results relative to the NGS profile detected.

### Endocrine and Reproductive Family Phenotypes

While in the proband the described complex interaction of multiple genetic alterations was associated with overt ovarian dysgenesis, infertility and ensuing need for hormonal replacement therapy, the patient's sister presented with normal pubertal development, achieving menarche at the age of 13 years, never reporting any clinical signs consistent with menstrual irregularity. As she requested safe contraception at the age of 19, she was started on estroprogestin pill, a therapy currently ongoing. However, when we assessed her residual ovarian function before prescribing hormonal therapy, we found decreased levels of anti-Müllerian hormone (AMH) (0.9 μg/L; reference value 1.5–13.3 μg/L), despite adequate gonadotropin (LH 4.5 U/L, FSH 7.0 U/L) and estradiol (42 pg/mL) levels and normal appearance of internal genitalia on pelvic US. This clinical picture was consistent with a diagnosis of diminished ovarian reserve (11), where a reduction in AMH concomitant with normal estradiol and gonadotropins levels may be predictive of progressive depletion of ovarian follicles, leading to a menopausal status in young adulthood. Conversely, the heterozygous variation of *FIGLA* found in the mother did not appear to have affected ovarian function, as she presented regular menses, with no clinical or biochemical signs of menopause at the age of 43 years—gonadotropins and AMH levels were in fact within the reference ranges for her age.

## Discussion

The present study reports on three family members (i.e., the proband and her sister and mother), carriers of one to three genetic variations, experiencing different degrees of residual ovarian function in a clinical spectrum potentially dependent on the number of genes and alleles involved. Specifically, the proband had early-onset ovarian dysgenesis associated with a homozygous variant of *FIGLA* together with heterozygous variants of *NOBOX* and *NR5A1* genes—the proband underwent hormonal therapy to induce puberty. An asymptomatic phenotype was instead observed for both the proband's mother, who carried a heterozygous variant of the *FIGLA* gene but retained normal ovarian function, and the proband's sister, who harbored heterozygous variants of both *FIGLA* and *NR5A1* genes but no signs of POI other than biochemical results consistent with diminished ovarian reserve. Importantly, except for the *NR5A1* variant, all the aforementioned genetic variants have not previously been described in the literature (ClinVar database).

The *FIGLA* gene encodes for a germ-cell specific transcription factor playing a major role in the regulation of multiple oocyte-specific genes, including those initiating folliculogenesis and those regulating the expression of various zona pellucida genes (12). Mutations involving this gene are mostly reported as responsible for POI with an autosomal dominant inheritance pattern (OMIM #612310) (13). Thus, it is somewhat surprising that in our family the POI phenotype was associated with a biallelic recessive loss-of-function of the *FIGLA* gene, an occurrence that, to date, has only been shown in two other families (14, 15).

Another important aspect emerging form this study is that the proband presented with three alterations of genes involved in oocyte differentiation and folliculogenesis (i.e., *FIGLA, NOBOX* and *NR5A1*). This, to the best of our knowledge, is the first report of a non-syndromic patient presenting with compound alterations of the aforementioned genes. However, despite the potential functional relevance of our genetic findings, we are well aware that from our data we cannot conclusively establish a causative link between gene expression of the three aforementioned variants and the overall endocrine and reproductive phenotype of the proband. It is in fact possible that the homozygous frameshift variant of the *FIGLA* gene alone may have been responsible for the early-onset POI, which would then call into question the synergic effect of the other genetic variants. Contrary to this view and in support to our central hypothesis, a previous NGS analysis of a panel of genes potentially involved in POI has shown that 42% of patients with POI may actually present with pathogenic variants of two or more genes involved in ovarian function or maturation (16). It is therefore possible that POI in patients with heterozygous variants in genes reported as causative factors for sporadic POI—identified through a traditional gene approach—may actually be the result of an interaction with additional variants involving different genes. Similar considerations have also been proposed by two other groups (14, 15), independently describing two couples of sisters with primary amenorrhea bearing a biologically inactive full-length FIGLA protein due homozygous missense mutations, but with unaffected heterozygous mothers. Moreover, the patients presented by Yuan et al. had short stature, while our proband's final height fell within normal Italian centiles (15). According to these authors, the normal ovarian function observed in these two mothers, as in our case, supported the hypothesis that *FIGLA* haploinsufficiency alone is not sufficient to cause POI. From this standpoint, overall in disagreement with other studies describing POI due to heterozygous mutations involving *FIGLA* (13), the different biochemical phenotypes that we have described in proband's mother and sister, harboring the same heterozygous *FIGLA* variant, could be regarded as the result of concomitant genetic variants. Even though from our data we cannot rule out that the phenotypic spectrum of the family described may be the result of an incomplete penetrance of the *FIGLA* variant, it is highly likely that the additional variants described in this study may act as modifier genes and, as such, may play a role in the pathogenesis and clinical severity of POI. However, as no other patients with this specific substitution have been reported so far, it is not quite possible at this stage to generalize our observation suggesting a cause-effect link between the expression of the two *NOBOX* and *NR5A1* heterozygous variants and POI.

The oocyte-specific homeobox gene encoding the newborn ovary homeobox protein (*NOBOX*) is expressed in germ cells and primordial oocytes. It belongs to a group of tissue-specific homeobox genes contributing to ovarian development. *NOBOX* is mostly expressed in primordial and growing oocytes and, when mutated, exerts a detrimental effect on ovarian function. Although pathogenic events have been most frequently associated with homozygous mutations in the *NOBOX* gene, a large study by Bouilly and colleagues has shown heterozygous mutation of the *NOBOX* gene (OMIM #610934) to be present in 5.6% of 213 unrelated patients with POI (17). In particular, the authors found five missense heterozygous variants with a detrimental effect on NOBOX transcriptional activity, even though functional studies ruled out a dominant-negative effect of those variants. On the other hand, two sisters described by França et al. (18) and a single patient reported by Li et al. (19) presented with POI due to a homozygous deletion involving this gene, with their heterozygous mothers being unaffected. In most patients reported, heterozygous mutations were associated with milder phenotypes, with spontaneous puberty progression, retrieved fertility, and premature menopause, while primary amenorrhea was mainly found in girls with homozygous variants (20). The presence of heterogeneous phenotypes associated with the same mutation in *NOBOX* as described in several cohorts suggests that also in this case no clear genotype-phenotype correlation can be defined for *NOBOX* variants. Although loss-of-function variants have been more frequently found in recessive forms and missense variants in dominant cases (18), more studies are warranted to confirm this association. It is possible in fact that additional detrimental variants involving different genes may result in POI in patients with *NOBOX* haploinsufficiency. In this regard, the reported *NOBOX* frameshift variant detected in our proband may have played a role in the precocious depletion of primordial oocytes, leading to ovarian dysgenesis in combination with the other reported variations.

Finally, although NR5A1 was initially considered as non-pathogenic in women given the maternal transmission of its mutated form in males with sex differentiation disorders (21), a growing body of literature has more recently demonstrated a potential role of this gene in ovarian insufficiency (22). NR5A1 is a nuclear receptor pivotal in the transcription of multiple target genes involved in adrenal and gonadal development, steroidogenesis, and reproduction. Homozygous mutations of this gene may alter both gonadal and adrenal steroidogenesis, leading to primary adrenal failure, while heterozygous mutations have been shown to cause POI in women (OMIM #612964) (21). The specific mutation found in our patient (p.Val355Met) had already been described by Philibert and colleagues in one boy with micropenis and testicular vanishing syndrome (21). As his mother, heterozygous for the same variant, had developed ovarian cysts and multiple miscarriages—potentially regarded as indirect signs of ovarian insufficiency—, it may be inferred that also heterozygous mutations in NR5A1 can mildly impair ovarian function. Although this conclusion cannot be systematically drawn from available data, it is tempting to speculate that heterozygous NR5A1 variant may play a detrimental role on ovarian function in both our proband and sister. Nevertheless, as in the family described this variant was inherited by both the daughters but absent in the mother, we hypothesize paternal inheritance. Intriguingly, the father had not been diagnosed with any specific gonadal issues. Probably, the ovarian impact of the reported genetic variation in NR5A1 will become clearer in the future, by comparing the reproductive and endocrine outcomes (i.e., age at onset of menopause, pregnancies) of the proband's sister to those of the mother. Indeed, as they share the same heterozygous *FIGLA* variant, an eventual deleterious effect of aberrant *NR5A1* would be proven in case of ovarian insufficiency and/or reproductive disorders in the sister, as the mother had achieved two pregnancies without any medically-assisted procreation techniques and had not presented any sign of menopause at the age of 43 years. The biochemical finding of suppressed AMH levels in the proband's sister may suggest a more compromised reproductive function. However, as *NR5A1* transcriptionally regulates AMH gene expression along with that of other transcription factors, the decreased AMH expression levels detected in the proband may not reflect the actual residual ovarian reserve.

## Conclusions

To the best of our knowledge, this is the first evidence in the literature of a family where a variable combination of three variants of genes involved in oocyte maturation may have played a synergic effect on clinically different phenotypes in relation to ovarian reserve and endocrine function.

Overall, this case outlines that the clinical phenotypic variability experienced by different patients carrying the same mutation may be the result of the detrimental effect of concurrent genetic factors.

## Ethics Statement

Written informed consent was obtained from the individual(s), and minor(s)' legal guardian/next of kin, for the publication of any potentially identifiable images or data included in this article.

## Author Contributions

AC conceived the idea of describing the present case report, drafted the paper, critically revised its contents and provided the final revision: in addition, AC produced the pictures of the paper. AT and AB drafted the first version of the paper. SM and AS performed the genetic counseling for the family and critically revised the final version of the paper. NM critically revised the contents of the paper and approved the final outcome. RR, LP, AD, and FG performed the genetic testing, analyzed the potential pathogenicity of the variants found, and actively contributed to the drafting of the paper. RR produced Table 1, for pathogenicity assessment of the genetic variants described, and Figure 3. All authors contributed to the article and approved the submitted version.

## Conflict of Interest

The authors declare that the research was conducted in the absence of any commercial or financial relationships that could be construed as a potential conflict of interest.
